# Genetic Differentiation, Niche Divergence, and the Origin and Maintenance of the Disjunct Distribution in the Blossomcrown *Anthocephala floriceps* (Trochilidae)

**DOI:** 10.1371/journal.pone.0108345

**Published:** 2014-09-24

**Authors:** María Lozano-Jaramillo, Alejandro Rico-Guevara, Carlos Daniel Cadena

**Affiliations:** 1 Laboratorio de Biología Evolutiva de Vertebrados, Departamento de Ciencias Biológicas, Universidad de los Andes, Bogotá, Colombia; 2 Department of Ecology & Evolutionary Biology, University of Connecticut, Storrs, Connecticut, United States of America; University of Arkansas, United States of America

## Abstract

Studies of the origin and maintenance of disjunct distributions are of special interest in biogeography. Disjunct distributions can arise following extinction of intermediate populations of a formerly continuous range and later maintained by climatic specialization. We tested hypotheses about how the currently disjunct distribution of the Blossomcrown (*Anthocephala floriceps*), a hummingbird species endemic to Colombia, arose and how is it maintained. By combining molecular data and models of potential historical distributions we evaluated: (1) the timing of separation between the two populations of the species, (2) whether the disjunct distribution could have arisen as a result of fragmentation of a formerly widespread range due to climatic changes, and (3) if the disjunct distribution might be currently maintained by specialization of each population to different climatic conditions. We found that the two populations are reciprocally monophyletic for mitochondrial and nuclear loci, and that their divergence occurred ca. 1.4 million years before present (95% credibility interval 0.7–2.1 mybp). Distribution models based on environmental data show that climate has likely not been suitable for a fully continuous range over the past 130,000 years, but the potential distribution 6,000 ybp was considerably larger than at present. Tests of climatic divergence suggest that significant niche divergence between populations is a likely explanation for the maintenance of their disjunct ranges. However, based on climate the current range of *A. floriceps* could potentially be much larger than it currently is, suggesting other ecological or historical factors have influenced it. Our results showing that the distribution of *A. floriceps* has been discontinous for a long period of time and that populations exhibit different climatic niches have taxonomic and conservation implications.

## Introduction

The limits of the geographic ranges of populations and species reflect the interplay of a variety of ecological and evolutionary forces such as migration, extinction and speciation [1]–[3]. Understanding how such forces underlie the origin and maintenance of disjunct distributions, in which closely related taxa or members of the same species occur in widely separate areas, is of central interest in biogeography [4], [5]. Hypotheses that may account for the disjunct distributions of species or close relatives include long-distance dispersal or the extinction of intermediate populations of a formerly continuous range, possibly as a result of geographic or climatic events, or human intervention. After disjunct distributions arise, the question becomes how are they maintained. Likely explanations for the maintenance of disjunct distributions are (1) environmental unsuitability of intervening areas and (2) adaptation to different environmental conditions in geographically separate areas [1], [6]–[11].

When historical distributions cannot be studied directly (i.e., using the fossil record), testing hypotheses about the origin of disjunct distributions can be accomplished using molecular phylogenetic estimates of divergence times between populations, which can be correlated with historical events [12]–[17]. This approach has provided insights into pervasive biogeographic patterns, such as the disjunct distribution of many organisms occurring in separate continents. For instance, based on the estimated time of lineage divergence, disjunct distributions of organisms occurring in America and Africa has been attributed to the split of Gondwana [17]–[20], transoceanic dispersal [21]–[23], human-mediated introductions [13], or various combinations of these processes [24].

Inferences about historical ranges and whether disjunct distributions might be the result of extinction of intermediate populations can also be made using ecological niche-modeling tools [25], [26] to generate historical estimates of potential species distributions based on climatic data [27]–[30]. For example, such models have indicated that some species with currently disjunct distributions may have been widely distributed in the past [29], [31]. If currently disjunct populations are relicts of more widespread lineages and one can construct models of the potential distributions at different times in the past, then one would expect to find a reduction in the connectivity between populations through time, with population separation matching the divergence dates estimated using molecular data. In addition, climatic data and statistical analyses based on null models can be used to evaluate the hypothesis that disjunct distributions are maintained at present time as a result of differentiation in climatic preferences between populations found in disjunct areas. Specifically, this hypothesis predicts that disjunct populations occur under different climatic environments as a result of niche divergence and that intervening areas are unsuitable for their occurrence [30].

**Figure 1:**
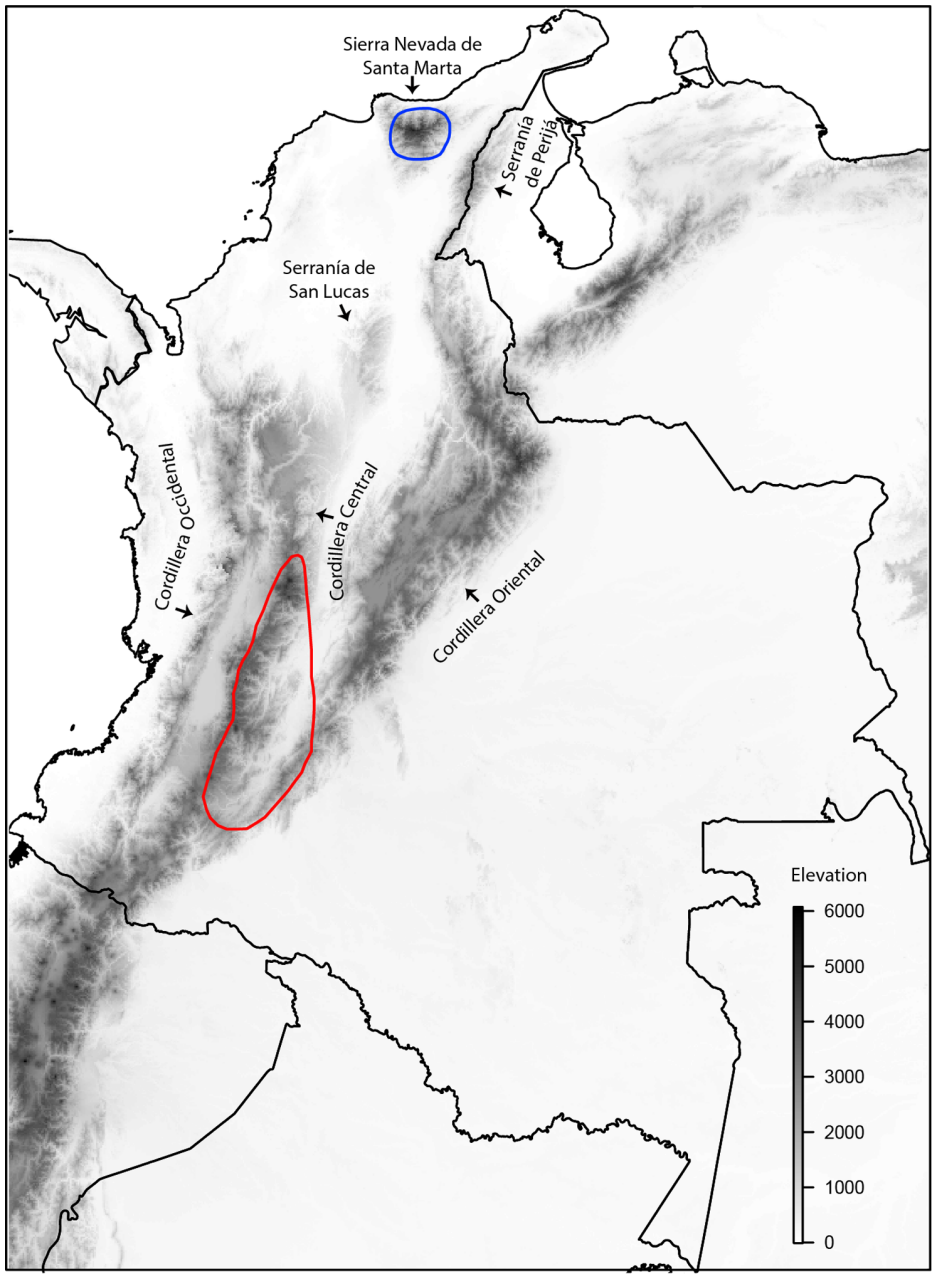
Current distribution of the Blossomcrown (*Anthocephala floricep*s). The blue area corresponds to *A. f. floriceps* from the Sierra Nevada de Santa Marta and the red to *A. f. berlepschi* from the Andes. The locations of different montane regions mentioned in the text are indicated.

The Blossomcrown (*Anthocephala floriceps* Gould, 1854), the single representative of a monotypic genus of hummingbird (Trochilidae) endemic to Colombia, is a good model in which to study disjunct distributions: two sedentary subspecies recognized based on plumage variation live in regions separated by more than 900 km (Fig. 1). *Anthocephala floriceps floriceps* is restricted to the foothills and mid elevations of the Sierra Nevada de Santa Marta in northern Colombia (500–1700 m), whereas *A. f. berlepschi* is found in the Andes (1200–2300 m) in Tolima and Huila departments [32]–[34]. In this study, we used DNA sequence data and niche modeling tools to (1) determine the timing of divergence between the two populations of *A. floriceps*, (2) assess whether the disjunct distribution of the species could have arisen as a result of fragmentation of a formerly widespread range owing to climate change over the Pleistocene, and (3) evaluate whether its disjunct distribution might be maintained by unsuitable intervening areas or specialization of each isolated population to different climatic conditions (niche divergence).

## Materials and Methods

### Molecular analyses

We used nuclear and mitochondrial DNA sequence data to examine genetic differentiation and to estimate the timing of divergence between populations of *A. floriceps.* These data allowed us to gain insight about factors potentially involved with the origin of their disjunct ranges. We extracted DNA from tissue samples of three museum specimens of *A. f. floriceps* and two of *A. f. berlepschi* (Table 1) using a phenol/chlorophorm protocol [35]. We then amplified and sequenced two mitochondrial (ND2 and ND4) and two nuclear genes (Bfib7 and ODC introns 6 and 7) for all individuals using published primers and protocols [36], [37]. We did not estimate gametic phase for the nuclear loci; apparent heterozygosities were coded as ambiguities using IUPAC codes. We combined our data (GenBank accession numbers KJ826445–KJ826464) with sequences of the same genes from three individuals of *A. f. berlepschi* obtained from GenBank (GU167208.1, GU166876, GU167098.1, GU166955.1; Table 1; [38]). As outgroups, we used four of the closest living relatives of *Anthocephala* identified by phylogenetic analyses of the Trochilidae [37], [39]. We obtained sequences for the ND2 and ND4 genes of the following outgroups from GenBank: *Campylopterus hemileucurus* (EU042534.1, EU042214.1), *Klais guimeti* (AY830495.1, EU042317.1), *Orthorhyncus cristatus* (AY830508.1, EU042328.1), and *Stephanoxis lalandi* (GU167250.1, GU166919.1).

**Table 1:** Specimens of *A. floriceps* included in molecular phylogenetic analyses.

Taxon	Tissue number	Locality
*A. f. floriceps*	ICN 36492	Santa Marta, Cuchilla de San Lorenzo
*A. f. floriceps*	ICN 36491	Santa Marta, Cuchilla de San Lorenzo
*A. f. floriceps*	ICN 36467	Santa Marta, Cuchilla de San Lorenzo
*A. f. berlepschi*	ANDES-BT 1311	Huila, Algeciras, Vereda Las Brisas, Finca Bélgica
*A. f. berlepschi*	ANDES-BT 1315	Huila, Algeciras, Vereda Las Brisas, Finca Bélgica
*A. f. berlepschi*	IAvH 1253	Huila, Palestina, Parque Nacional Natural Cueva de los Guácharos
*A. f. berlepschi*	IAvH 1269	Huila, Palestina, Parque Nacional Natural Cueva de los Guácharos
*A. f. berlepschi*	IAvH 1255	Huila, Palestina, Parque Nacional Natural Cueva de los Guácharos

ICN: Instituto de Ciencias Naturales, Universidad Nacional de Colombia; ANDES-BT: Banco de Tejidos, Museo de Historia Natural de la Universidad de los Andes; IAvH: Instituto Alexander von Humboldt.

To estimate the divergence time between the two populations of *A. floriceps*, we constructed a chronogram in BEAST 1.5.2 [40] based on a concatenated matrix including sequences of both mitochondrial genes for the two populations and outgroups. We conducted this analysis using the HKY+G substitution model, which was selected as the best fit to the data according to the Akaike Information Criterion (AIC) in ModelTest 3.7 [41]. To calibrate our tree based on analyses including ND4 data, we used the ND2 substitution rate of 2.5% divergence per million years [42], and related the corrected distances for ND2 with the distances obtained combining ND2 and ND4 data using a linear regression. Because the slope of the regression was 1.11 (r^2^ = 0.99), we multiplyed the ND2 per-lineage rate of 0.0125 by 1.11, and fixed the product (0.0139) as the mean rate for calibration. We fitted a relaxed molecular clock with lognormal rate-variation, and ran 50 million generations sampling every 1000 steps and discarding the first 10,000 as burn-in. We used TRACER v1.5 to check that effective sample sizes of parameter estimates were greater than 200. As an additional way to examine relationships among mtDNA and nucDNA sequences, we also constructed haplotype networks (for concatenated mitochondrial data and separately for each nuclear locus) using the median-joining algorithm in the software Network 4.5.1.6 [43].

### Ecological niche modeling

We first used ecological niche modeling tools to (1) determine whether areas located in between the two disjunct distribution ranges of *A. floriceps* are unsuitable for its occurrence, and (2) to assess whether the distribution of *A. floriceps* could have been more widespread in the past (i.e., at different periods in the Pleistocene). For these analyses, we used 43 localities obtained from museum specimens ([44], Global Biodiversity Information Facility (GBIF: http://www.gbif.org)), field observations (N. Gutiérrez, pers. comm.), and published data [34]. We characterized each locality with 19 climatic variables at 1 km x 1 km resolution obtained from WorldClim [45]; these variables are commonly used in ecological niche modeling and indicate annual trends, seasonality, and extreme values in temperature and precipitation. We considered all of Colombia and western Venezuela and generated a model of the potential distribution of *A. floriceps* in this area at present using the maximum enthropy algorithm implemented in Maxent 3.3.2 [27]. We used default settings to obtain a logistic model output with continuous values ranging from 0 to 100, with higher values indicating greater probabilities of occurrence. Following model-validation using the area under the receiver-operating-characteristic (ROC) curve and a binomial test of omission [27], we projected the model onto climate layers for 6,000 years before present (ybp), the Last Glacial Maximum (LGM; aprox. 21,000 ybp), and 130,000 ybp [45], [46]. To distinguish climatically suitable from unsuitable sites, we applied the “fixed cumulative value 10” threshold rule in Maxent [47]. We visually assessed the extent of potential distributions at these different time periods.

We also used ecological niche modeling to evaluate whether the currently disjunct distribution of *A. floriceps* might be maintained by specialization of each population to different climatic conditions. To accomplish this, we first modeled the potential distribution at present of each population separately using the 19 climatic variables. We then projected models generated for each population onto the geographic region where the other population occurs to assess whether each model would classify the localities where the other population has been recorded as climatically suitable (i.e., model interprediction). Low model interprediction would support the hypothesis of climatic specialization maintaining disjunct ranges. However, because the two populations occur in geographically distinct areas where climate may differ considerably irrespective of the presence or absence of the study species, lack of interprediction of distribution models does not necessarily reflect intrinsic niche divergence between populations; populations may have equivalent fundamental niches yet occupy different environments (i.e., different realized niches) due solely to geographic differences in climate [10], [48], [49]. Thus, we sought to determine whether the environments where populations occurred were more or less similar that expected by chance based on differences in the climatic conditions of the regions within which the ranges of each population are embedded. To do so, we examined climatic divergence between populations relative to a null divergence model using the climatic background of the range of each population, an approach that allows for explicit testing of niche divergence vs. niche conservatism [10]. For this analysis we used the 19 WorldClim variables and also elevation; we reduced these 20 variables using a principal component analysis (PCA) and then employed the first four principal components (accounting for c. 97% of the variance, see below) as observed niche values. To establish background variation in climate, we extended polygons depicting the known distribution range of each population of *A. floriceps* [50] 20 km in all directions and randomly placed 1000 points within each expanded polygon. Values for elevation and the 19 climatic variables were extracted for all of these points. Niche divergence and conservatism were assessed by comparing the observed difference in mean niche values to the difference in mean background (i.e., null) values for each of the four principal components. Niche divergence, i.e., specialization to different climates, as a potential factor accounting for the maintenance of disjunct distributions would be supported if population niches were more divergent than expected based on background divergence [10]. Tests were conducted in R version 2.12.2 [51].

## Results

### Molecular analyses

**Figure 2:**
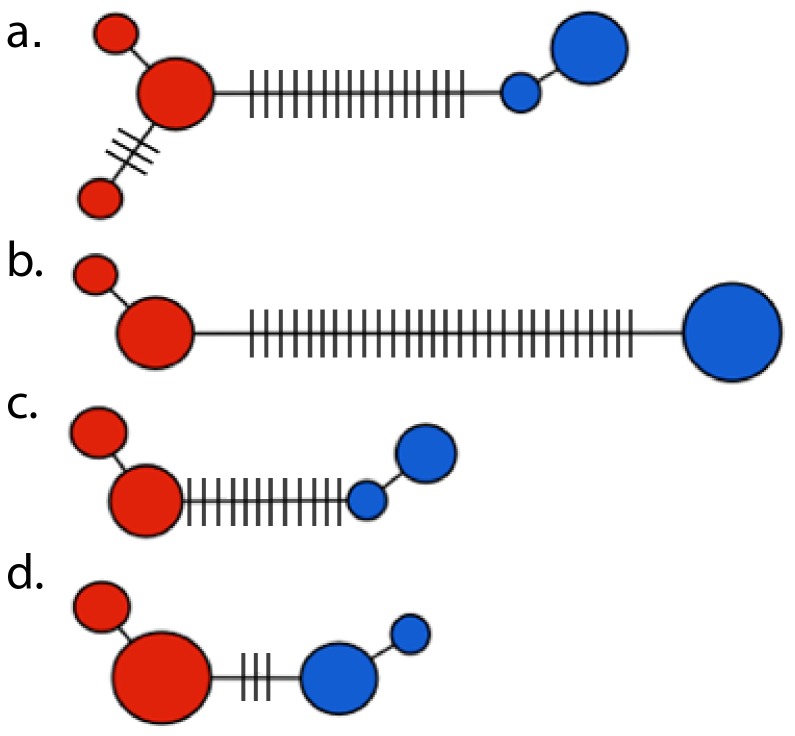
Haplotype networks showing that no alleles are shared between populations of *A. floriceps* in any of the genes analyzed. Blue corresponds to *A. f. floriceps* and red to *A. f. berlepschi*. Circle size is proportional to the number of individuals with each haplotype; hatches indicate mutational steps. (a) ND2, (b) ND4, (c) Bfib7 and (d) ODC.

**Figure 3:**
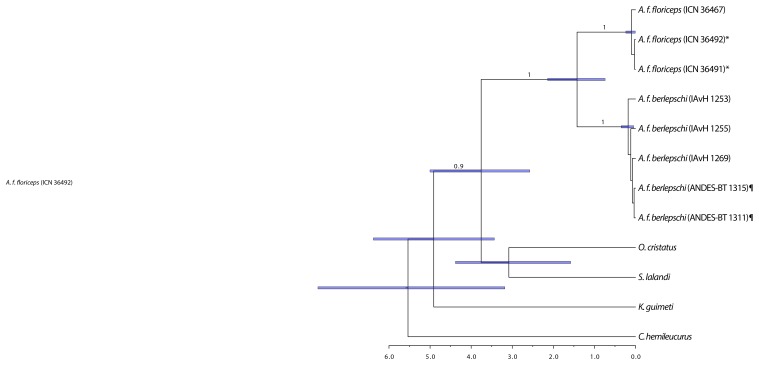
Divergence-time estimates (mya) between populations of *A. floriceps* and outgroups, based on two mitochondrial genes using a Bayesian relaxed molecular-clock analysis. Node bars indicate 95% credibility intervals on node ages; scale bar shows time in million years. Values on each clade indicate posterior probabilities when greater than 0.7. Symbols indicate individuals having identical sequences in *A. f. floriceps* (*) and *A. f. berlepschi* (¶).

Genealogies showed the same pattern for all genes: each subspecies of *A. floriceps* formed a monophyletic group comprising distinct haplotypes (Fig. 2). Although our sample sizes were low, this pattern suggested the two populations have been isolated for a considerable time span, long enough to have achieved reciprocal monophyly in both mitochondrial and nuclear loci. Furthermore, the chronogram based on mtDNA sequences indicated that the two subspecies were reciprocally monophyletic groups whose divergence dates to c. 1.4 million years before present (mybp; 95% credibility interval 0.7–2.1 mybp; Fig. 3).

### Ecological niche modeling

**Figure 4:**
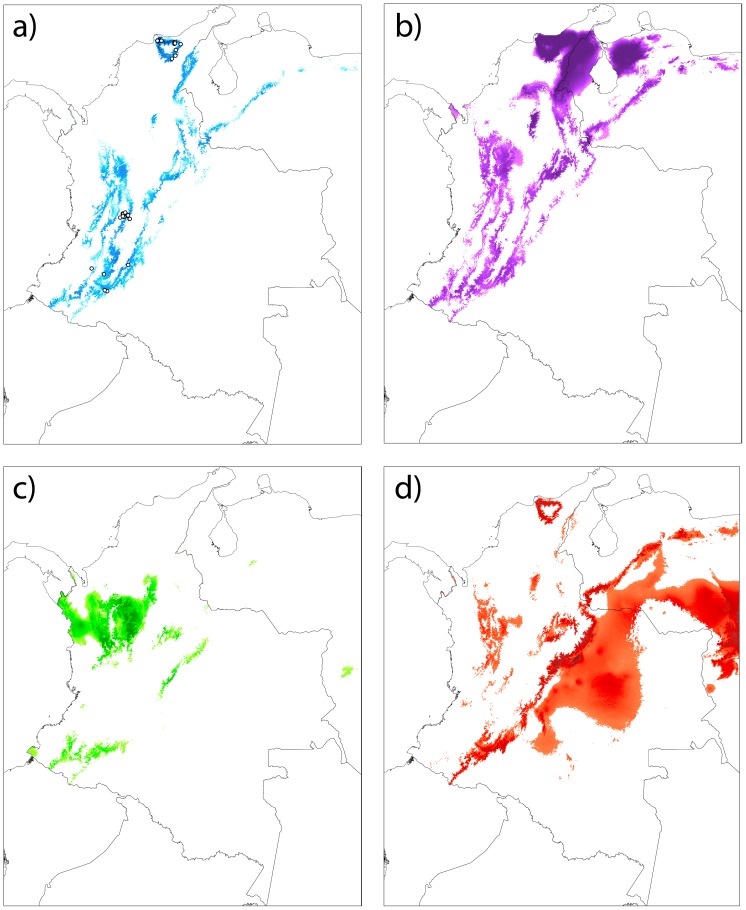
Potential distributions for *A. floriceps* predicted using climatic data in Maxent. Models are shown for climatic conditions of (a) the present, (b) 6,000 ybp, (c) 21,000 ybp and (d) 130,000 ybp. Dots on the present distribution map indicate localities used to build the models. Darker colors denote areas of greater climatic suitability; areas in white are below the minimum suitability threshold and are therefore considered to be unsuitable.

The area under the ROC curve for the model predicting the potential distribution of *A. floriceps* at present was close to one (0.983), indicating it performed substantially better than chance. Additionally, the binomial test of omission was significant (p< 0.001), suggesting that the species’ distribution was adequately predicted based on climate. This model suggested that environmental conditions suitable for the occurrence of *A. floriceps* existed well beyond the boundaries of its current range in the Andes (Fig. 4a). This indicates that, based on the climatic variables studied, at least part of the range disjunction cannot be attributed to climatic unsuitability of intervening areas.

Because the model based on climatic data adequately predicted the present-day distribution (i.e., point localities) of *A. floriceps*, assuming niche conservatism one can use such models to examine the potential distribution of the species in the past based on historical climate. None of the historical distribution ranges estimated by the model were sufficiently large suggesting there was potential for the species to be continuously distributed in the past (Fig. 4). However, the potential distribution for 6,000 ybp was considerably larger and more continuous than the potential distribution at present (Fig. 4b); at this time, the Sierra Nevada de Santa Marta appears to have been connected to the northern end of the Cordillera Oriental of the Andes (i.e., Serranía de Perijá) by areas suitable for the presence of *A. floriceps* across the intervening lowlands. Moreover, environments potentially suitable for the species appear to have been more extensively distributed in the northern sector of the Cordillera Central and in the Serranía de San Lucas and surrounding lowlands 6,000 ybp relative to the present. In contrast, much of the area now occupied by *A. floriceps* (including all of the range of *A. f. floriceps* in the Sierra Nevada de Santa Marta) appear to have been unsuitable for the species 21,000 ybp (Fig. 4c). Finally, for 130,000 ybp, the model identified continuous areas of potentially high climatic suitability along the eastern slope of the Cordillera Oriental and extending into lowland areas east of the Andes, but revealed no potential connections between the currently disjunct populations (Fig. 4d).

**Figure 5:**
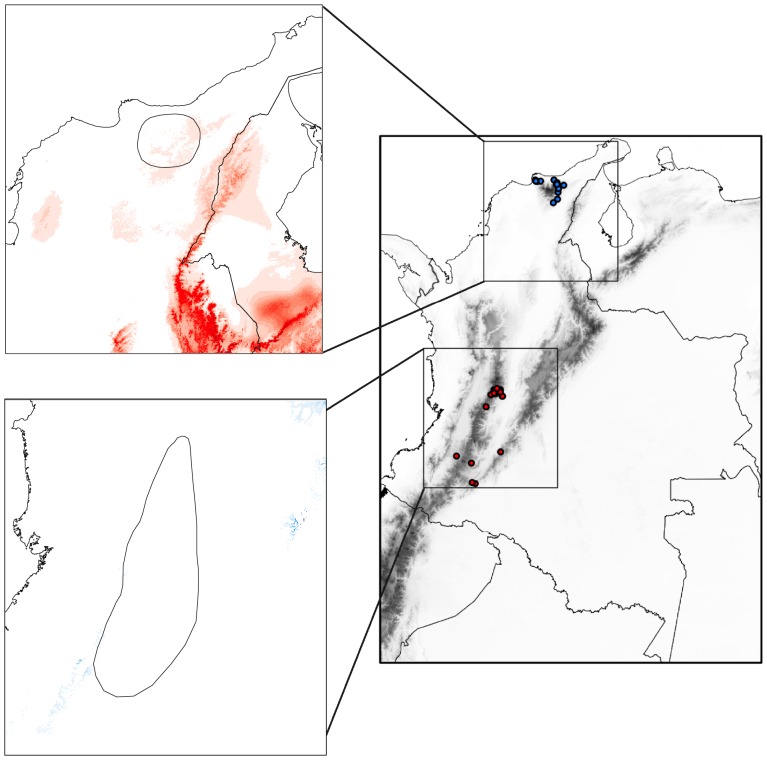
Model of potential distribution constructed based on localities of *A. f. berlepschi* projected onto the region where *A. f. floriceps* occurs (indicated by a blue shape; (a)). Model of potential distribution constructed based on localities of *A. f. floriceps* projected onto the region where *A. f. berlepschi* occurs (indicated by a red shape; (b)). Red and blue dots indicate localities used to build the models for *A. f. berlepschi* and *A. f. floriceps,* respectively. Darker colors denote areas of greater climatic suitability in a continuous scale (i.e., no cutoff threshold was established in Maxent). Note that localities of each population have low suitability according to the model constructed with data from the other population, indicating niche divergence.

Potential distribution models constructed separately for each population based on present-day climate also had area under ROC curves close to one (*A. f. floriceps*: 0.981, *A. f. berlepschi*: 0.944). However, the distribution model constructed for each population did not predict the current distribution of the other (Fig. 5), implying that each population inhabits environments with different climatic conditions. This result was supported by tests of niche divergence and conservatism (Table 2). The axis explaining most of the variation (PC1; 40%) was largely associated with elevation and temperature and was the only one revealing significant niche conservatism. The other three axes (jointly accounting for c. 57% of environmental variation) revealed significant niche divergence between populations associated with precipitation and seasonality (Table 2; Table S1). The Andean population lives in less humid and less seasonal environments than the population from the Sierra Nevada de Santa Marta (Fig. S1).

**Table 2:** Divergence on niche axes between populations of *A. floriceps.*

	Niche axes				
	PC1	PC2	PC3	PC4	
**Pairwise comparison**					
*A. f. floriceps* vs *A. f. berlepshi*	**0.78C**	**1.23 D**	**0.70 D**	**1.37 D**	
	(0.58, 1.26)	(0.30, 1.55)	(0.63, 0.89)	(1.13, 2.31)	
Variance explained (% )	40%	24%	22%	11%	
Top four variable loadings	elevation*	bio16	bio17*	bio3*	
	bio6	bio13	bio14*	bio14	
	bio11	bio12	bio12*	bio15	
	bio10	bio18	bio18	bio17*	

Instances of significant niche divergence (D) or conservatism (C) are shown in bold (t-test; p<0.05). Values in parentheses represent the 95% confidence intervals of the null distributions based on background divergence between the geographic ranges of each population. For each niche axis, the top four environmental variables loading on it are shown (asterisks indicate opposite sign). bio3 = isothermality, bio6 = minimum temperature of coldest month, bio10 = mean temperature of warmest quarter, bio 11 = mean temperature of coldest quarter, bio12 = annual precipitation, bio13 = precipitation of wettest month, bio14 = precipitation of driest month, bio15 = precipitation seasonality, bio16 = precipitation of wettest quarter, bio17 = precipitation of driest quarter, bio18 = precipitation of warmest quarter. For full results of principal components analysis see Fig. S1.

## Discussion

### The origin and maintenance of the disjunct distribution in *A. floriceps*

Our estimates of potential distributions based on climatic data indicated that in four time periods over the last 130,000 ybp, including the present, climatic conditions have likely not been suitable for *A. floriceps* to have had a fully continuous distribution. The only possible exception to this pattern is the inferred connection between the Sierra Nevada de Santa Marta and the northern stretches of the Cordillera Oriental (Serranía de Perijá) suggested by the predicted potential distribution for 6,000 ybp (Fig. 4b). Also at 6,000 ypb, the species appears to have had a more extensive potential distribution along the Cordillera Central, which may have allowed for connectivity between this mountain range, the Serranía de Perijá and the Sierra Nevada de Santa Marta via the Serranía de San Lucas and surrounding areas, a region in which climatically suitable areas appeared to have been considerably more extensive than at present (Fig. 4). If either scenario is correct, then the species must have gone extinct not only from the lowland environments separating the Sierra Nevada de Santa Marta from the Perijá, but also from the full extent of the Perijá, the Serranía de San Lucas and the Cordillera Oriental, mountain systems where it does not presently exist.

We note, however, that estimates of potential historical distributions based on ecological niche modeling must be considered cautiously because the realized conditions under which species exist at present (i.e., those used to build ecological niche models) may not fully represent their fundamental niches and could lead to potentially misleading reconstructions of their geographic ranges at other times. Especially in scenarios where combinations of climatic conditions that existed in the past are not equivalent to those existing in the present, i.e., non-analogous climates, models based only on present-day conditions may not accurately estimate historical distributions [52], [53]. We suspect this likely applies to our estimate of potential distribution for *A. floriceps* at 21,000 ybp, when its potential range appeared to have been substantially reduced, with no suitable environments in the Sierra Nevada de Santa Marta, the region where one of its present-day populations is endemic (Fig. 4c). Based on patterns of genetic variation indicating marked distinctiveness of the Santa Marta population (see below), that the species was absent from this mountain range at this time and colonized it subsequently seems unlikely.

Because GIS layers depicting estimates of historical climate in our study region are unavailable for dates earlier than those we examined, we cannot address the possibility that the range of *A. floriceps* became disjunct at an earlier moment in history using ecological niche modeling. Can molecular data provide insights about the origin of its disjunct distribution? Our molecular-clock analysis suggests that the divergence between mtDNA clades dates to c 1.4 million mybp (credibility interval 0.7–2.1 mybp), suggesting that divergence occurred prior to the period for which historical climate data are available. However, we note that the inferred timing of divergence reflects gene divergence, which may be considerably older than population/taxon divergence [54], [55]. This is a likely possibility considering that Neotropical montane birds often show strong population genetic differentiation even along continuous ranges [56], [57].

Our results are consistent with the hypothesis that the currently disjunct distribution of *A. floriceps* may persist due to specialization of each isolated population to different climatic conditions. Ecological niche models suggest that populations of *A. floriceps* are divergent in their climatic niches beyond what one would expect given the climatic background where they exist, implying that a plausible explanation for the maintenance of their disjunct ranges is climatic niche divergence. It makes sense that both populations exhibit a conserved niche axis related to elevation and temperature because their elevational ranges overlap broadly [34]. However, our analyses revealed significant niche divergence in relation to precipitation and seasonality, with the Andean population occupying less humid and less seasonal environments. If this reflects that each population is adapted to specific climatic conditions and not simply that realized climatic conditions differ between regions but fundamental climatic niches do not, then climatic restrictions likely do not allow the species’ geographic distribution to become fully continuous [10], [48], [49], [58]–[60].

Although our models failed to reveal continuous potential distributions in the past and at present and populations showed significant climatic divergence, climatic unsuitability of intervening areas and niche divergence between populations are not sufficient explanations for the c. 900-km discontinuity in the present-day range of *A. floriceps.* The modeled potential distribution at present (Fig. 4a) indicates that environmental conditions suitable for its occurrence exist through much of the Cordillera Central of the Colombian Andes, a region lacking obvious environmental discontinuities [2]. Also, suitable conditions exist along the western slope of the Cordillera Oriental albeit with some notable environmental breaks (Fig. 4a; [2]). Thus, based on climatic conditions, the distribution range of *A. floriceps* could potentially be larger than it currently is, especially in the Cordillera Central. A similar result was obtained in a recent study examining disjunct populations of Painted Buntings (*Passerina ciris*) in North America, where areas not occupied by the species were found to be potentially suitable for its occurrence [30]. The restricted distribution range of the Andean form *A. f. berlepschi* likely reflects ecological factors not accounted for by climatic variation (e.g., biotic interactions) or historical factors limiting range expansion. The influence of historical factors is likely, considering *A. f. berlepschi* is one of several members of a distinctive assemblage of codistributed taxa restricted to an area of endemism in the departments of Tolima and Huila [8], [61], [62].

### Taxonomic and conservation implications

Our divergence time estimates between populations of *A. floriceps* (1.4 mybp) suggest an older date than the reported divergence times for phylogroups within some Neotropical hummingbird species [60], [63]–[65] and even between several lineages recognized as different species of hummingbirds [66]. Our analyses further showed that subspecies do not share haplotypes in four different genes including nuclear loci, with their four-fold higher coalescence times relative to mtDNA, indicating long-term isolation without gene flow. We realize our sample sizes are not large enough to provide a robust test of reciprocal monophyly, but given the strong divergence and geographic isolation, we suspect our conclusions would be robust to analyses with larger sample sizes.

In conclusion, our data suggest that the current distribution of *A. floriceps* has been disjunct for a relatively long time. Furthermore, each population occurs under distinct climatic conditions, which likely reflects evolved differences in their climatic niche. Our results revealing strong genetic and climatic divergence between populations of *A. floriceps*, together with morphological differences that led to their recognition as different subspecies, arguably have taxonomic implications. The evidence for marked divergence and reciprocal monophyly in mitochondrial and nuclear loci, in addition to climatic differentiation and morphological diagnosability, implies that each population could be considered a full species under several species concepts [67]–[71]. Applying the criterion of reproductive isolation central to the biological species concept is impossible owing to the allopatric distributions of the two populations, but divergence in several respects between them, relative to divergence between “good” species of hummingbirds [72], may suffice to consider them to be reproductively isolated [73]. In any event, the likelihood that the two forms may eventually come into contact appears extremely unlikely, so their status as independently evolving units will most likely be maintained and should probably prevail in terms of establishing their taxonomic status [74]. At the very least, our work shows that these populations are divergent lineages meeting the criteria for recognition as evolutionarily significant units worthy of attention from a conservation standpoint and requiring independent management [75], [76]. Their distinctiveness has likely been overlooked as a consequence of traditional taxonomy treating them as conspecific, a situation that may apply to several other populations of Neotropical birds with disjunct ranges [77].

## Supporting Information

Figure S1 **Bivariate plots showing climatic differences between localities occupied by** ***Anthocephala floriceps floriceps*** **in the Sierra Nevada de Santa Marta (blue) and** ***A. f. berlepschi*** **in the Andes (red).** Note that *A. f. berlepschi* occurs in drier areas with more stable temperature and less seasonal precipitation than *A. f. floriceps.*
(TIF)

Table S1 **Variables used to characterize the ecological niches of populations of** ***Anthocephala floriceps*** **and their loadings on the first four axes obtained following principal components analyses.** These four axes accounted for 97% of the variation. The variables with the four highest loadings on each principal component are shown in bold.
(DOCX)
