# Correction: Genetic Differentiation, Niche Divergence, and the Origin and Maintenance of the Disjunct Distribution in the Blossomcrown *Anthocephala floriceps* (Trochilidae)

**DOI:** 10.1371/journal.pone.0116065

**Published:** 2014-12-16

**Authors:** The PLOS ONE Staff

There is an error in Figure 3. Please see the corrected version of Figure 3 here.

**Figure 3:**
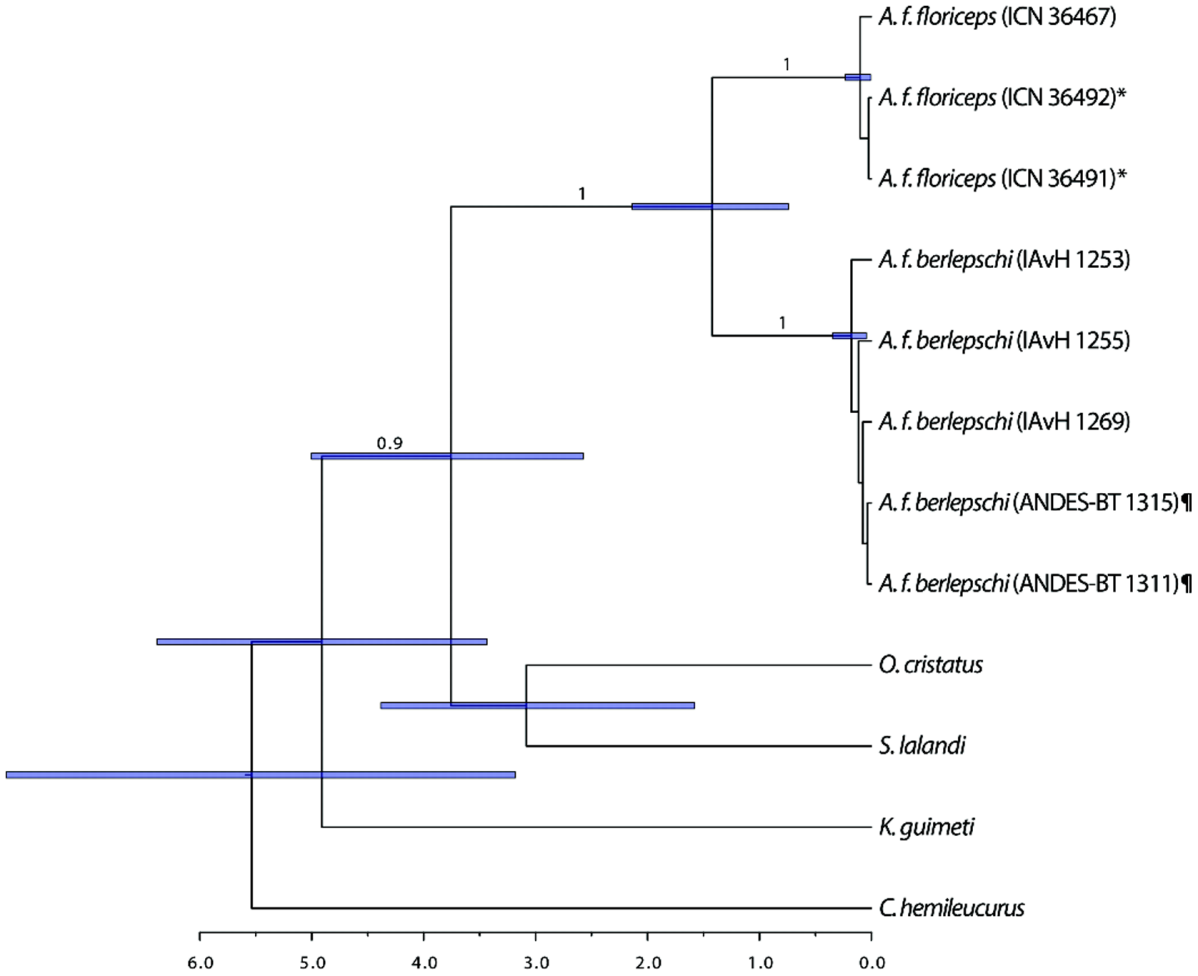
Divergence-time estimates (mya) between populations of *A. floriceps* and outgroups, based on two mitochondrial genes using a Bayesian relaxed molecular-clock analysis. Node bars indicate 95% credibility intervals on node ages; scale bar shows time in million years. Values on each clade indicate posterior probabilities when greater than 0.7. Symbols indicate individuals having identical sequences in *A. f. floriceps* (*) and *A. f. berlepschi* (¶).

## References

[1] Lozano-Jaramillo M, Rico-Guevara A, Cadena CD (2014) Genetic Differentiation, Niche Divergence, and the Origin and Maintenance of the Disjunct Distribution in the Blossomcrown *Anthocephala floriceps* (Trochilidae). PLoS ONE 9(9): e108345 doi:10.1371/journal.pone.0108345 25251766 10.1371/journal.pone.0108345 PMC4176958

