# Supplementary figures and images for: Genetic Differentiation, Niche Divergence, and the Origin and Maintenance of the Disjunct Distribution in the Blossomcrown Anthocephala floriceps (Trochilidae)
Source: PLoS One. 2014 Sep 24;9(9):e108345. doi: 10.1371/journal.pone.0108345 (PMC4176958; doi:10.1371/journal.pone.0108345)

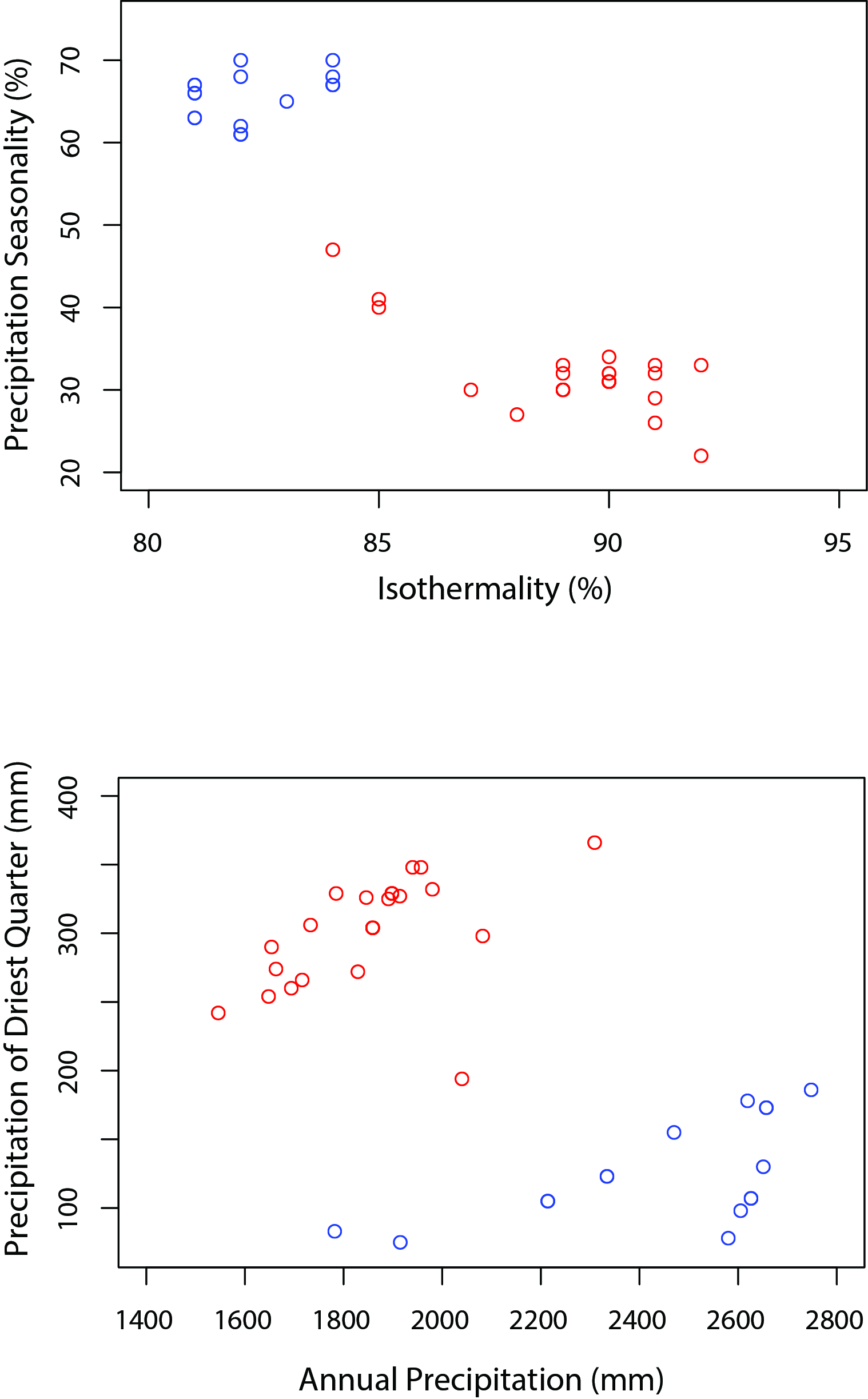

Supplement: Figure S1 — Bivariate plots showing climatic differences between localities occupied by Anthocephala floriceps floriceps in the Sierra Nevada de Santa Marta (blue) and A. f. berlepschi in the Andes (red). Note that A. f. berlepschi occurs in drier areas with more stable temperature and less seasonal precipitation than A. f. floriceps. (TIF) [file pone.0108345.s001.tif]
