# Supplementary material for: Genetic Differentiation, Niche Divergence, and the Origin and Maintenance of the Disjunct Distribution in the Blossomcrown Anthocephala floriceps (Trochilidae)
Source: PLoS One. 2014 Sep 24;9(9):e108345. doi: 10.1371/journal.pone.0108345 (PMC4176958; doi:10.1371/journal.pone.0108345)
Supplement: Table S1 — Variables used to characterize the ecological niches of populations of Anthocephala floriceps and their loadings on the first four axes obtained following principal components analyses. These four axes accounted for 97% of the variation. The variables with the four highest loadings on each principal component are shown in bold. (DOCX) [file pone.0108345.s002.docx]

|  | **PC1** | **PC2** | **PC3** | **PC4** |
| --- | --- | --- | --- | --- |
| Elevation | **-0.91** | 0.20 | -0.24 | -0.41 |
| bio1 = Annual mean temperature | 0.85 | -0.20 | 0.23 | 0.36 |
| bio2 = Mean diurnal temperature range | 0.34 | -0.03 | 0.63 | 0.07 |
| bio3 = Isothermality | -0.35 | -0.10 | -0.15 | **-0.83** |
| bio4 = Temperature seasonality | 0.51 | -0.03 | -0.01 | 0.74 |
| bio5 = Maximum temperature of warmest month | 0.84 | -0.18 | 0.35 | 0.37 |
| bio6 = Minimum temperature of coldest month | **0.89** | -0.24 | 0.09 | 0.31 |
| bio7 = Annual temperature range | 0.42 | 0.01 | 0.81 | 0.38 |
| bio8 = Mean temperature of wettest quarter | 0.87 | -0.22 | 0.21 | 0.36 |
| bio9 = Mean temperature of driest quarter | 0.80 | -0.21 | 0.74 | 0.33 |
| bio10 = Mean temperature of warmest quarter | **0.88** | -0.20 | 0.72 | 0.39 |
| bio11 = Mean temperature of coldest quarter | **0.89** | -0.21 | 0.24 | 0.33 |
| bio12 = Annual precipitation | -0.32 | **0.90** | **-0.86** | -0.23 |
| bio13 = Precipitation of wettest month | -0.08 | **0.96** | 0.07 | 0.14 |
| bio14 = Precipitation of driest month | -0.46 | 0.23 | **-0.88** | **-0.80** |
| bio15 = Precipitation seasonality | 0.53 | 0.07 | 0.24 | **0.77** |
| bio16 = Precipitation of wettest quarter | -0.16 | **0.97** | 0.01 | 0.10 |
| bio17 = Precipitation of driest quarter | -0.47 | 0.34 | **-0.96** | **-0.76** |
| bio18 = Precipitation of warmest quarter | -0.21 | **0.84** | -0.14 | -0.27 |
| bio19 = Precipitation of coldest quarter | -0.33 | 0.61 | **0.84** | -0.34 |
